# Potential impact of great lockdown on students’ knowledge, attitude and practices towards the COVID-19 outbreak

**DOI:** 10.12688/f1000research.110874.1

**Published:** 2022-05-03

**Authors:** Mahmoud Berekaa, Eltigani Omer, Munthir Almoslem, Khaled Alsahli, Mohammed Aljassim

**Affiliations:** 1 Environmental Health Department, College of Public Health, Imam Abdulrahman Bin Faisal University, Dammam, Eastern Province, Dammam 31441, Saudi Arabia; 2 Public Health Department, College of Public Health, Imam Abdulrahman Bin Faisal University, Dammam, Eastern Province, Dammam 31441, Saudi Arabia

**Keywords:** COVID-19 pandemic, preparatory-year students, perception, non-pharmaceutical intervention, Knowledge, attitudes, behavior, preventive measures, IAU students

## Abstract

**Background** **:** Despite variation in the types of COVID-19 vaccines and genetic variation in the SARS-CoV-2 genome, using preventive measures remains the first choice to reduce risks associated with COVID-19 infection.

**Methods:** This cross-sectional study highlights students’ knowledge, attitudes, and practices toward SARS-CoV-2 infection during the lockdown. The study was conducted at the Imam Abdulrahman Bin Faisal University (IAU), Eastern Province, Saudi Arabia. Data was collected from 43 male preparatory students via an online self-structured questionnaire. Knowledge level was determined using mean scores, while chi-square and t-tests were performed to detect significant differences between groups.

**Results:** Males aged 17–20 displayed better knowledge regarding COVID-19 than other groups (t = 2.03, p = 0.049). Most participants recognized the typical symptoms and transmission routes; 93% indicated they viewed social distancing as a crucial preventive measure. Following lockdown, a 1.7-fold increase in the number of participants who believed that mask-wearing was an effective preventive measure was observed; however, 37.2% did not trust this practice. There was a 2.2-fold increase in the number of participants who took part in awareness programs during lockdown. Students’ knowledge increased from 73.2% to 81.5% following the lockdown. Specifically, mean knowledge regarding the role of contaminated tools in disease transmission increased from 33.7% to 75.5%. After lockdown, 58.1% of participants were anxious and afraid of having contact with their colleagues, while 39.5% missed their classes due to anxiety. Two sources of information impacted students’ knowledge following the lockdown: university studies (t = 2.149, p = 0.038) and friends (t = 2.6, p = 0.013).

**Conclusions:** The findings suggest that IAU preparatory-year students have acceptable knowledge, awareness, and attitudes towards COVID-19 infection. This reflects the impact of media on the improvement in preventive measure uptake. Knowledge of the pandemic may increase if health education programs are designed to target students.

## Introduction

The emergence of the novel COVID-19 coronavirus disease has become a global health concern. COVID-19, caused by SARS-CoV-2, was first detected in Wuhan city, China. ^1^^–^^4^ The most significant risk associated with COVID-19 is its potential to cause a severe acute response that may result in death. Epidemiological studies have revealed that the virus is spread, transmitted, and gains host entry by various means. ^2^^,^^5^^–^^8^ COVID-19 has directly impacted numerous sectors, such as public health, the economy, and education. During the early stages of the outbreak, higher education institutions responded in different ways, with some having strong reactions that disproportionally impacted students who experience anxiety. For example, students from the Imam Abdulrahman Bin Faisal University (IAU) responded to the unprecedented preventive measures that were enforced by the Saudi Arabian government to control the spread of COVID-19. However, students’ responses to non-therapeutic protective measures are significantly influenced by their knowledge, behavior, and attitude toward the disease. While poor understanding and risk perception of COVID-19 among community sectors, such as educational institutes and healthcare providers, may significantly increase disease spread and infection levels, ^9^^–^^11^ high knowledge and awareness levels among students can limit the disease spread. ^12^ Baloran ^13^ reported that non-therapeutic interventions should be highly effective during a pandemic. In a study conducted during the MERS-CoV pandemic, Al-Mohaissen ^14^ reported that most Saudi Arabian university communities were aware of the disease’s epidemiology and symptoms; however, there was less awareness regarding the preventive measures that could limit its spread. Olaimat ^15^ reported that university students in Jordan had adequate knowledge regarding COVID-19 infection, with significant levels of knowledge observed among postgraduate students using the internet, social media, and mass media as sources of COVID-19 information.

In early March 2020, Saudi Arabia announced its first COVID-19 case. Subsequently, all on-premise educational activities, including at IAU, were suspended, with all activities becoming virtual. ^16^ Several studies have been conducted on medical and non-medical university students in Saudi Arabia, including during the lockdown period, to explore their knowledge, awareness, and attitudes towards COVID-19. ^17^^–^^20^ On a community level, major precautions to combat the spread of the virus were taken by several countries, including Saudi Arabia. Several researchers have attempted to monitor the citizens’ knowledge levels and behaviors during lockdown periods. ^21^^–^^27^

This research aims to determine the impact of the lockdown on IAU students’ knowledge regarding COVID-19’s transmission methods and symptoms and their attitudes and practices regarding preventive measures during their study. Emphasis was placed on the impact of social media and family as sources of COVID-19 knowledge and awareness. Furthermore, this study documents the behavior changes of IAU students during the lockdown period, particularly their interaction with infected or individuals suspected of being infected, with a focus on student anxiety.

## Methods

### Study location

The study involved preparatory year students from IAU, Eastern Province, Saudi Arabia. The study took place before and after the lockdown period associated with COVID-19, which was between the second academic semester in March 2020 and the first academic semester in October 2020.

### Sampling design and methods

The study population primarily consisted of 43 randomly selected preparatory male students enrolled in health specialties. The sample size was calculated using Cochran’s formula. ^28^ Baseline data pertaining to the participants’ pre-lockdown COVID-19 awareness and practices was gathered. Following lockdown, another sample of male students was randomly selected, and the impacts of lockdown on their awareness of COVID-19 were measured. A randomization policy was used to avoid bias in the selection of the study units.

The IAU QuestionPro platform was used for data collection. A pre-validated structured online questionnaire was constructed, generated, and randomly distributed to the students. The same questionnaire was used to assess knowledge levels and attitudes both pre- and post-lockdown.

### Statistical analysis

The data was processed and analyzed using the SPSS19.0 program (SPSS Inc., Chicago, IL, USA). A descriptive and mean difference analysis questionnaire was conducted, with the percentage of responses to each section calculated. A statistical test was used to determine the presence of significant changes in the knowledge and attitudes of the students toward COVID-19. All variables were matched during the analysis to remove any confounding variables.

### Ethical consideration

Ethical permission was obtained from the Institutional Review Board (IRB) of IAU Dammam, Saudi Arabia (IRB-2021-03-020). Written and verbal consent was obtained from all participants.

## Results

### Students’ characteristics

Demographic data indicated that, before lockdown, participants aged 17–20 years and 21–23 years made up 69.8% and 20.9% of the sample, respectively. After lockdown, those same age groups made up 88.4% and 11.6% of the sample, respectively. The number of household members each participant had was measured before lockdown, with 3–5, 6–8, and 9 or more household members making up 27.9%, 48.8%, and 14% of the sample, respectively.

Table 1 demonstrates the differences in participants’ COVID-19 knowledge with regard to their demographic characteristics. There are no statistically significant differences between knowledge level and age group in the period before the lockdown. However, a statistically significant difference in knowledge and age group was seen after lockdown. Participants aged 17–20 demonstrated greater knowledge than other groups (t = 2.03, p = 0.049). Regarding the number of household members, there was a statistically significant difference in the participants’ knowledge scores based on the number of household members they had.

**Table 1.** Knowledge differences based on demographic characteristics.

Variable	N	Before lockdown Mean ± SD	After lockdown Mean ± SD	
**Age**				
17–20 years	30	74.8	38	82.3
21–23 years	9	69.7	5	75.0
t	1.382		2.03	
p	0.859		0.049 *	
**Household members**				
3–5	12	72.1	NA ^	
6–8	21	73.7		
9 or more	6	71.6		
F	0.151			
p	0.860			

* Statistically significant.

^ NA: not available.

### Knowledge about COVID-19

Excluding one, all participants had heard about COVID-19 prior to the lockdown; 90.7% of them knew it was a virus and the main causative agent of COVID-19. Following lockdown, 100% of participants had heard of it and knew it as the main causative agent.

Regarding methods of transmission of the COVID-19 ( Table 2), the most commonly recognized methods pre-lockdown were sneezing (86%), touching (65.1%), hugging (41.9%), coughing (69.8%), and shaking hands (60.5%). Post-lockdown, improvement in knowledge levels around transmission methods was observed, with 97.7%, 95.3%, 79.1%, 97.7%, and 97.7% of participants recognizing the aforementioned methods, respectively. Additionally, the average knowledge regarding contaminated tools, doorknobs, money, and mobile phone as methods of disease transmission increased from 33.73% to 75.48%, pre- and post-lockdown, respectively.

**Table 2.** Knowledge regarding COVID-19 methods of transmission.

Item	Before lockdown		After lockdown	
	Yes	No	Yes	No
	N (%)	N (%)	N (%)	N (%)
Sneezing	37 (86)	6 (14)	42 (97.7)	1 (2.3)
Toughing	28 (65.1)	15 (34.9)	41 (95.3)	2 (4.7)
Hugging	18 (41.9)	25 (58.1)	34 (79.1)	9 (20.9)
Coughing	30 (69.8)	13 (30.2)	42 (97.7)	1 (2.3)
Shaking hands	26 (60.5)	17 (39.5)	42 (97.7)	1 (2.3)
Contaminated tools	23 (53.5)	20 (46.5)	35 (81.4)	8 (18.6)
Doorknobs	16 (37.2)	27 (62.8)	37 (86)	6 (14)
Money	14 (32.6)	29 (67.4)	35 (81.4)	8 (18.6)
Mobile phone	5 (11.6)	38 (88.4)	23 (53.5)	20 (46.5)

Knowledge levels about the major sources of COVID-19 infection are depicted in Table 3. The correct answers were air and person-to-person contact. Pre-lockdown, 41.9% of students correctly identified air as an infection source, while 81.4% correctly identified person-to-person contact. However, 55.8% wrongly considered contaminated meat to be a source of infection, and 32.6% considered domestic animals to be. Furthermore, some students incorrectly identified water, soil, cross-contamination with camels, and seafood as infection sources.

**Table 3.** Knowledge regarding sources of COVID-19 infection.

Item	Before lockdown		After lockdown	
	Yes	No	Yes	No
	N (%)	N (%)	N (%)	N (%)
The major source of infection is:				
Water	2 (4.7)	41 (95.3)	2 (4.7)	41 (95.3)
Air	18 (41.9)	25 (58.1)	23 (53.5)	20 (46.5)
Soil	3 (7)	40 (93)	2 (4.7)	41 (95.3)
Person-to-person contact	35 (81.4)	8 (18.6)	42 (97.7)	1 (2.3)
Contaminated meat	24 (55.8)	19 (44.2)	4 (9.3)	39 (90.7)
Cross-contamination with domestic animals	14 (32.6)	29 (67.4)	6 (14)	37 (86)
Cross-contamination with birds	7 (16.3)	36 (83.7)	4 (9.3)	39 (90.7)
Cross-contamination with camels	6 (14)	37 (86)	4 (9.3)	39 (90.7)
Seafood	3 (7)	40 (93)	0 (0)	43 (100)

Recognition of air and person-to-person contact as infection sources greatly improved following lockdown, with 53.5% of students correctly identifying air as an infection source and 97.7% identifying person-to-person contact. However, some students did still cite other incorrect infection sources in their answers.

Table 4 displays the participants’ knowledge regarding the main COVID-19 symptoms. The correct symptoms were fever, cough, shortness of breath, and headaches. Pre-lockdown, many students correctly identified fever (79.1%), cough (76.7%), and shortness of breath (72.1%) as COVID-19 symptoms. However, just over a third (34.9%) of the students considered headaches a symptom, while other methods, such as runny nose, diarrhea, and pain, were identified incorrectly.

**Table 4.** Knowledge of COVID-19 symptoms.

Item	Before lockdown		After lockdown	
	Yes	No	Yes	No
	N (%)	N (%)	N (%)	N (%)
The main symptoms of the disease are:				
Fever	34 (79.1)	9 (20.9)	43 (100)	0 (0)
Cough	33 (76.7)	10 (23.3)	42 (97.7)	1 (2.3)
Shortness of breath	31 (72.1)	12 (27.9)	43 (100)	0 (0)
Runny nose	22 (51.2)	21 (48.8)	16 (37.2)	27 (62.8)
Diarrhea	12 (27.9)	31 (72.1)	22 (51.2)	21 (48.8)
Headache	15 (34.9)	28 (65.1)	36 (83.7)	7 (16.3)
Joint pain	3 (7)	40 (93)	16 (37.2)	27 (62.8)

After lockdown, all students correctly identified fever and shortness of breath as symptoms, and 97.7% correctly identified a cough as a symptom. Additionally, headaches were recognized as a symptom by 65.1% of students. However, runny nose, joint pain, and diarrhea were mentioned by some participants still.

Table 5 demonstrates participants’ knowledge regarding COVID-19 protection methods. Before lockdown, a low level of knowledge regarding the correct protection methods was observed among participants. However, this increased following the lockdown. For example, 79.1% of students correctly identified “avoid contact with infected persons” as a protection method pre-lockdown, while 100% identified it post-lockdown. Furthermore, despite mask-wearing being an effective preventive measure, only 37.2% trusted this practice pre-lockdown. However, this increased to 62.8% following lockdown. Moreover, fewer students identified the incorrect statement “avoid contact with domestic animals” as a protection method following lockdown, with the proportion of responses decreasing from 25.6% to 14%.

**Table 5.** Knowledge about COVID-19 protection measures.

Item	Before lockdown		After lockdown	
	Yes	No	Yes	No
	N (%)	N (%)	N (%)	N (%)
To protect yourself you should:				
Avoid contact with infected persons	34 (79.1)	9 (20.9)	43 (100)	0 (0)
Avoid spending a long time outdoors	23 (53.5)	20 (46.5)	37 (86)	6 (14)
Avoid crowded areas/places	24 (55.8)	19 (44.2)	40 (93)	3 (7)
Regular handwashing	29 (67.4)	14 (32.6)	43 (100)	(0)
Cook healthy food	5 (11.6)	38 (88.4)	7 (16.3)	36 (83.7)
Avoid hugging	10 (23.3)	33 (76.7)	37 (86)	6 (14)
Visit a physician if you show any symptoms of the disease	27 (62.8)	16 (37.2)	31 (72.1)	12 (27.9)
Use disposable masks	16 (37.2)	27 (62.8)	27 (62.8)	16 (37.2)
Avoid contact with domestic animals	11 (25.6)	32 (74.4)	6 (14)	37 (86)
Avoid eye and ear contacts	17 (39.5)	26 (60.5)	40 (93)	3 (7)

### Knowledge levels regarding COVID-19 infection

**Figure 1.**
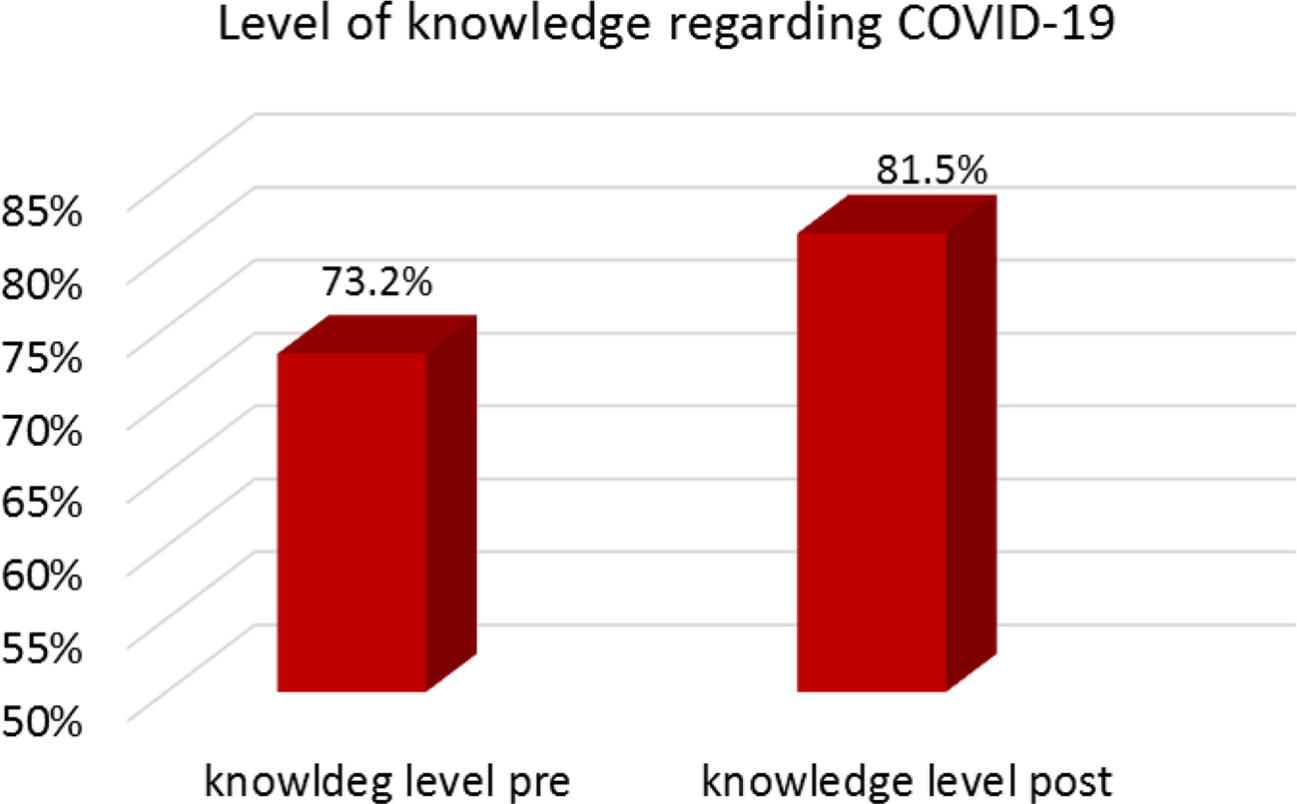
Students’ knowledge levels regarding COVID-19 infection.

Figure 1 demonstrates participants’ knowledge levels regarding COVID-19 pre- and post-lockdown. Pre-lockdown, the mean knowledge score was 73.2%. Post-lockdown, this increased to 81.5%.

**Figure 2.**
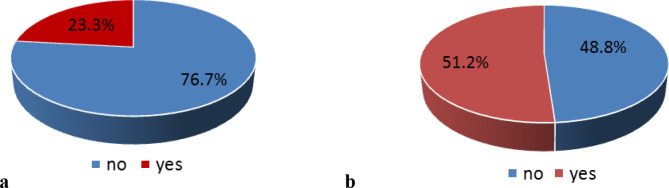
Participants who received information on COVID-19 infection: before lockdown (a) and after lockdown (b).

Figure 2a and 2b show the distribution of the participants who received information about COVID-19. Pre-lockdown, 23.3% of participants had received information on the disease. Post-lockdown, this increased to 51.2%.

**Figure 3.**
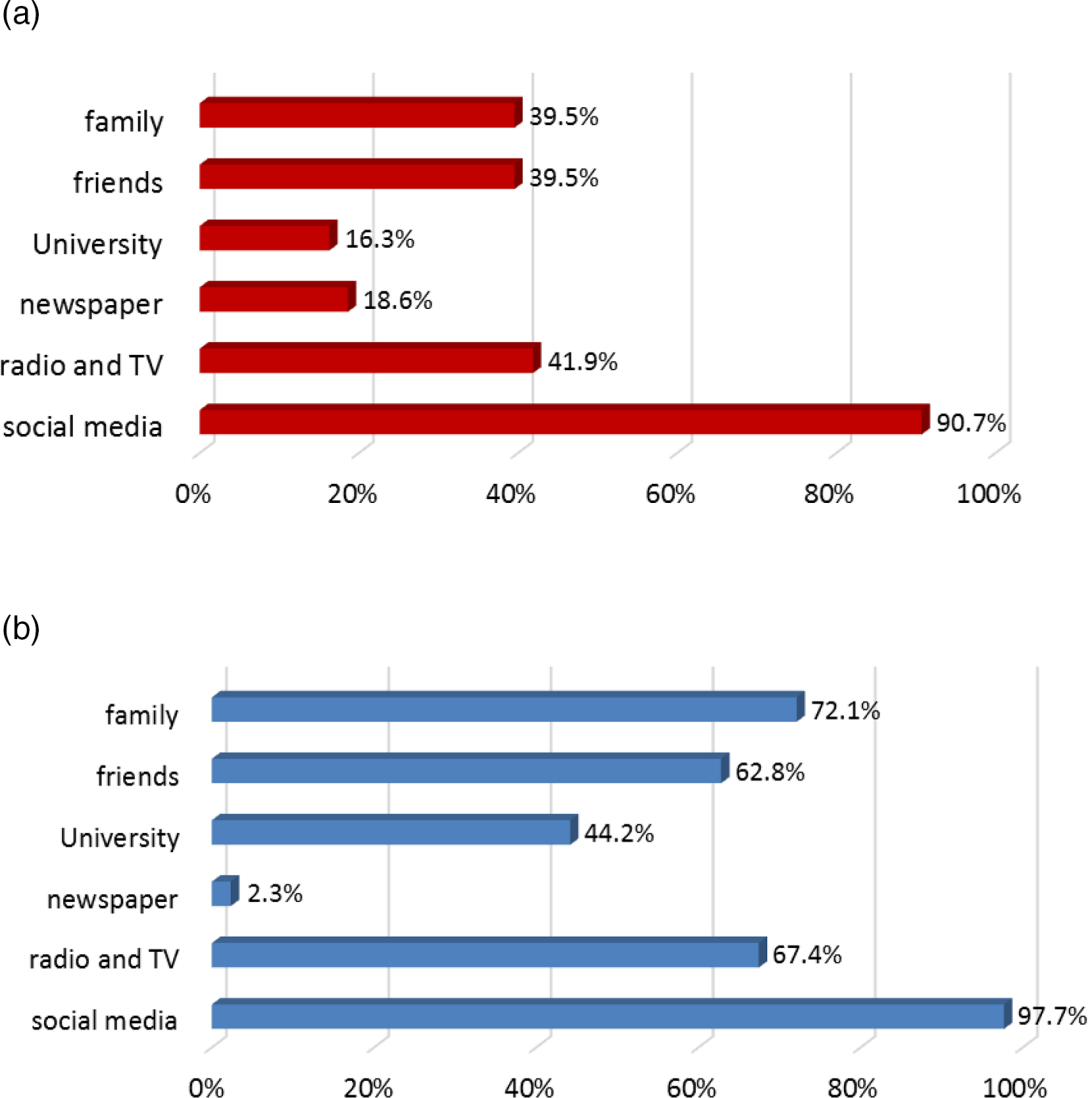
a: Sources of information regarding COVID-19 infection before lockdown. b: Sources of information regarding COVID-19 infection after lockdown.

Figure 3a and 3b depict the sources of information used by students regarding COVID-19. All sources of information increased in use post-lockdown except for newspaper. University studies contributed to the awareness of16.3% of students pre-lockdown and 44.2% post-lockdown.

Regarding information sources and their effects on participants’ knowledge, Table 6 shows no statistically significant differences between knowledge and any information sources pre-lockdown. However, two sources of information significantly affected participants’ knowledge post-lockdown; university studies (t = 2.149, p = 0.038) and friends (t = 2.6, p = 0.013).

**Table 6.** Differences between participants’ knowledge regarding sources of information.

Variable	N	Before lockdown Mean ± SD	N	After lockdown Mean ± SD
Did you get your information about the disease from social media?				
Yes	39	74±10.4	42	81.3±7.9
No	4	65.6±5.9	1	87.5±0
t	2.462		-0.777	
p	0.055		0.441	
Did you get your information about the disease from radio & TV programs?				
Yes	18	75±11.1	29	81.6±8.5
No	25	71.9±9.7	14	81.1±6.4
t	0.970		0.220	
p	0.338		0.827	
Did you get your information about the disease from newspapers?				
Yes	8	73.1±10.7	1	70±0
No	35	73.2±10.4	42	81.7±7.7
t	-0.022		-1.501	
p	0.983		0.141	
Did you get your information about the disease from university?				
Yes	7	76.1±12.5	19	83.68+8.3
No	36	72.6±10	24	78.7±6.8
t	0.801		2.149	
p	0.428		0.038*	
Did you get your information about the disease from friends				
Yes	27	75.1±11.1	2.611	83.7±8.2
No	16	71.9±9.8	2.611	77.7±5.5
t	1.001		2.611	
p	2.6		0.013 *	
Did you get your information about the disease from family?				
Yes	17	73.1±10.4		82.7±8.5
No	26	73.3±10.5		78.1±4.7
t	-0.056		1.777	
p	0.956		0.083	

* Significant at p<0.05.

### Knowledge regarding vaccinations and drug treatments

Participants’ knowledge regarding the COVID-19 vaccine revealed that pre-lockdown, 11.6% were aware of the vaccination, while 27.9% and 60.5% responded with “no” and “I don’t know,” respectively. In contrast, post-lockdown, 14% responded with “yes,” while 60.5% and 25.6% responded with “no” and “I don’t know,” respectively. Moreover, regarding knowledge about the presence of a COVID-19 drug, pre-lockdown, only 23.3% of the participants were aware of its presence, while 27.9% and 48.8% responded with “no” and “I don’t know,” respectively. Post-lockdown, 41.9% responded with “yes,” while 44.2% and 14% responded with “no” and “I don’t know,” respectively.

### Attitudes regarding COVID-19

Table 7 demonstrates participants’ attitudes toward COVID-19. Pre-lockdown, 39.5% of participants were anxious and afraid of coming into contact with their IAU colleagues due to the disease. However, only one was absent from class due to anxiety. Post-lockdown, 58.1% of participants were anxious and afraid of coming into contact with their colleagues, and 39.5% missed classes due to anxiety.

**Table 7.** Participants’ attitudes toward COVID- 19 infection.

Item	Before lockdown		After lockdown	
	Yes	No	Yes	No
	N (%)	N (%)	N (%)	N (%)
Are you anxious/afraid of contact with your IAU colleagues due to the disease (during the pandemic)?	17 (39.5)	26 (60.5)	25 (58.1)	18 (41.9)
During the early stages of the pandemic, were you absent from the classes due to anxiety related to the disease?	1 (2.3)	42 (97.7)	17 (39.5)	26 (60.5)

### Practices

Table 8 shows participants’ practices regarding COVID-19, with the incorrect answers being “avoid contact and offer no help” and “help with no concern for safety measures.” The participants showed increased levels of correct practice post-lockdown. Regarding the practice of helping a suspected or infected person while using safety measures, the percentage of correct responses increased from 53.5% pre-lockdown to 76.7% post-lockdown. Likewise, correct responses toward sticking with the strict protection measures increased from 53.5% to 90.7%, while contacting the relevant organizations increased from 65.1% to 90.7%. Regarding the incorrect practice statements, most students responded correctly with “no” post-lockdown.

**Table 8.** Participants’ practices regarding COVID- 19 infection.

Item	Before lockdown		After lockdown	
	Yes	No	Yes	No
	N (%)	N (%)	N (%)	N (%)
If you encounter a suspected or infected person, will you:				
Avoid contact and offer no help	4 (9.3)	39 (90.7)	4 (9.3)	39 (90.7)
Help with no concern for safety measures	2 (4.7)	41 (95.3)	2 (4.7)	41 (95.3)
Help but apply strict safety and protective measures	23 (53.5)	20 (46.5)	33 (76.7)	10 (23.3)
Stick with strict protection measures	23 (53.5)	20 (46.5)	39 (90.7)	4 (9.3)
Contact the relevant organization/s	28 (65.1)	15 (34.9)	39 (90.7)	4 (9.3)

## Discussion

Protection against a highly contagious disease, such as COVID-19, requires strict adherence to guidelines and rules, particularly regarding non-therapeutic interventions. Application of such measures requires adequate knowledge, attitudes, and practices on an individual and community level. ^12^^,^^17^^,^^23^ The present study assessed the influence of lockdowns on IAU students’ awareness levels during the COVID-19 pandemic. The majority of the participants, who were preparatory year students, were aged between 17–20 and 21–23 years old. As young students are considered to be typically asymptomatic carriers and tend to have more social lifestyles, they may play a significant role in the dissemination of the disease, particularly among their colleagues, family, and friends. ^8^^,^^12^

Assessment of participants’COVID-19 knowledge indicated that knowledge regarding different methods of COVID-19 disease transmission (e.g., sneezing, touching, and coughing) greatly improved following lockdown. The role of coughing and sneezing in disseminating the virus has been reported by several health agencies and organizations. ^29^^,^^30^ This increase in knowledge demonstrates the crucial role played by Saudi authorities in disseminating this knowledge during lockdown. ^23^

The participants in this study showed a good understanding that water, soil, meat, contact with domestic animals, camels, and seafood are not involved in the infection process. However, misconceptions about the cause of COVID-19 have been reported among other students. ^29^^,^^30^^,^^12^ All participants acknowledged that good personal hygiene and regular handwashing were important ways to protect themselves from COVID-19. Khasawneh et al. ^31^ indicated that maintaining good personal hygiene and regular handwashing are the first lines of defense against COVID-19. IAU students demonstrated increased awareness levels regarding social distancing, avoiding hugging, wearing masks, and limiting person-to-person contact and increased knowledge of COVID-19 symptoms post-lockdown, supporting the international regulations and national precaution preventive measures that were put in place during lockdown. ^1^^,^^2^^,^^7^^,^^12^^,^^30^^,^^32^^–^^34^ In a study assessing COVID-19 knowledge, attitude, and practices among the public of Saudi Arabia, approximately half of the respondents were unaware that COVID-19 could spread from person to person via aerosols. ^23^ However, a study in Qassim found that 90.1% of participants were aware of person-to-person transmission. ^18^ Moreover, in other studies, 90% of undergraduate students in China and 79% of undergraduate students in Indonesia acknowledged the role of respiratory droplets in disease transmission and increased infection risk. ^3^^,^^35^

In the current study, the participants demonstrated significant knowledge levels regarding the three major COVID-19 symptoms. Post-lockdown, fever and shortness of breath were correctly identified by all participants, while cough was identified by almost all. Similar results have been observed among the Saudi Arabian public, Saudi Arabian medical interns, Chinese undergraduate students, and in the Qassim region, with 94.75%, 96%, 98.6%, and 97.4% of respondents in these groups correctly identifying coughing as a symptom. ^18^^,^^19^^,^^23^^,^^35^ Additionally,89.7% Indonesian undergraduate students correctly identified cough and fever as COVID-19 symptoms. ^3^

Knowledge regarding COVID-19 drugs and vaccines was satisfactory among the current study participants; only a small number believed there was a vaccine against the infectious agent. Similarly, 96% of the Saudi Arabian public knew there was no clinically approved treatment for COVID-19. ^23^ Unfortunately, during the time of the current study, Singh et al. ^12^ reported that 92% of students were aware that no vaccine exists for COVID-19.

Social media and TV programs demonstrated significant impacts on the dissemination of knowledge and information regarding COVID-19 infection post-lockdown. Furthermore, the influence of social relationships with family and friends in providing COVID-19 information increased improved by 2.3-fold and 1.5-fold, respectively, post-lockdown. The influence of friends was particularly significant among 17–20-year-olds. Similarly, the role of IAU in improving students’ knowledge increased 2.7-fold, particularly among 17–20-year-olds. As expected, newspapers were not significantly used as sources of information pre- or post-lockdown.

Similarly, a cross-sectional study by Sobaih et al. ^37^ cited the impact of social media usage during COVID-19 and its importance in promoting social learning among students from nine public higher education institutions in underdeveloped countries. In a case study, Hashim et al. ^38^ reported that social media, particularly television broadcasts, were viewed as the most trusted source of information among approximately 147 students from a Malaysian technical university. A cross-sectional study analyzing the COVID-19 knowledge, attitudes, and practices of students from the University of Sharjah, United Arab Emirates, reported that the internet and social media were major sources of information for 85.2% of health-related and non-health-related students. ^39^ Khasawneh et al. ^31^ carried out a cross-sectional study involving students from six medical schools in Jordan, revealing that social media (83.4%) and online research engines (84.8%) were the preferred sources of COVID-19 information among the students. The preference of students to share verified COVID-19 information with family and friends via social media, which can be considered a third-party method of information, is an interesting finding.

The current study’s results indicated that the influence of family and friends in providing COVID-19 information increased following lockdown. However, mutual knowledge exchange between students and their families may occur, particularly regarding COVID-19 information. ^12^ Saud et al. ^40^ noted the importance of social media platforms as ab easy and accessible way to disseminate COVID-19 information between family and friends.

The current study revealed that IAU students experienced increased levels of anxiety due to COVID-19, particularly around having contact with IAU colleagues. Furthermore, an increased level of absenteeism was observed following lockdown. On the contrary, students in Japan were less anxious due to their adherence to precautionary behaviors. ^41^ The impacts of lockdown on students’ mental health and the need for psychological support have been reported by many researchers. ^42^^–^^48^ The assessment of IAU students’ responses toward suspected or infected persons indicated a high level of awareness regarding the importance of following strict safety and protective measures. However, research on how individuals react towards other individuals with COVID-19 is scarce; this is an area that requires further investigation.

## Conclusions

The frequent variation in the COVID-19 genome has led to the requirement of different vaccine types, reflecting the importance of preventive measures in mitigating the risks associated with COVID-19 infection. Students’ lack of knowledge regarding the nature of the disease and the precautions that should be taken may have increased disease spread. The findings of the current study suggest that IAU preparatory students had acceptable levels of knowledge, awareness, and attitudes toward COVID-19 infection. The results highlight the impact of media on the improvement of preventive measure uptake. Students’ COVID-19 knowledge may be significantly improved if a proactive health education program is designed for them. The work presented in this study can serve as the basis for the construction of awareness programs that can act as efficient non-therapeutic interventions for students with limited basic pandemic knowledge.

## Data availability

### Underlying data

Figshare: COVID-19 after.sav. DOI: https://doi.org/10.6084/m9.figshare.19390571 ^49^

This study contains the following underlying data:

- COVID19 After.sav (Two main files; COVID-19 Outbreak.sav and COVID19 After.sav contain participants responses data before and after lockdown period.
- Data comprises; gender, age, family members, college and department, knowledge about the disease, attitudes and practices about the COVID-19 disease).
- All datasets have been de-identified in accordance with Safe Harbour Method.

Data are available under the terms of the Creative Commons Attribution 4.0 International license (CC-BY 4.0).
