# Supplementary material for: Potential impact of great lockdown on students’ knowledge, attitude and practices towards the COVID-19 outbreak
Source: F1000Res. 2023 Jul 17;11:484. Originally published 2022 May 3. [Version 2] doi: 10.12688/f1000research.110874.2 (PMC10366551; doi:10.12688/f1000research.110874.2)
Supplement: Peer review history

# Peer review: Reviewer response for version 2

**DOI:** 10.5256/f1000research.152804.r187809

**Published:** 2023-07-24

**Authors:** Alok Atreya

**Affiliations:** 1 Lumbini Medical College, Palpa, Nepal

I would like to extend my appreciation for your prompt and comprehensive response to the reviewers' comments. I have carefully reviewed the revised manuscript and your point-by-point responses, and I am pleased to inform you that the manuscript has improved significantly in light of the revisions made.

 I am satisfied with the modifications you made to the manuscript to address the concerns raised during the initial review. The inclusion of years after 17-20 in the results section, the detailed description of the development and validation of the online self-structured questionnaire, and the clarification on how written informed consent was obtained have notably enhanced the rigor and transparency of the study.

 I also acknowledge the addition of the section discussing the limitations of the study, which helps contextualize the findings and provides valuable insights for readers.

 Furthermore, the manuscript's adherence to the IMRAD structure, the logical flow from introduction to conclusion, and the clear presentation of the work demonstrate the high quality of your research. Additionally, I am satisfied with the technical soundness of the study design and the availability of all source data underlying the results, ensuring full reproducibility.

 Regarding the occasional grammatical errors, I am glad to hear that the manuscript has been subjected to proofreading before submission. This will undoubtedly enhance the readability and overall presentation of your work.

 After careful consideration, I am pleased to approve the manuscript for indexing in F1000 research. Your study contributes significantly to our understanding of students' knowledge, attitudes, and practices during the COVID-19 pandemic and will be a valuable addition to the current literature.

 Once again, I commend you and your co-authors for your diligent work and thank you for choosing F1000 Research for the dissemination of your research.

 Thank you for your commitment to advancing academic research.

Is the work clearly and accurately presented and does it cite the current literature?

Yes

If applicable, is the statistical analysis and its interpretation appropriate?

I cannot comment. A qualified statistician is required.

Are all the source data underlying the results available to ensure full reproducibility?

Yes

Is the study design appropriate and is the work technically sound?

Yes

Are the conclusions drawn adequately supported by the results?

Yes

Are sufficient details of methods and analysis provided to allow replication by others?

No

Reviewer Expertise:

Forensic Medicine & Toxicology, and broad areas of interest are injuries, violence, trauma, abuse, medical ethics, medical education, forensic pathology, forensic anthropology, criminal law, forensic psychiatry, etc.

I confirm that I have read this submission and believe that I have an appropriate level of expertise to confirm that it is of an acceptable scientific standard.

# Peer review: Reviewer response for version 1

**DOI:** 10.5256/f1000research.122528.r155351

**Published:** 2023-07-05

**Authors:** Alok Atreya

**Affiliations:** 1 Lumbini Medical College, Palpa, Nepal

The manuscript investigates the impact of the COVID-19 lockdown on the knowledge, attitudes, and practices of preparatory-year students at Imam Abdulrahman Bin Faisal University (IAU) in Saudi Arabia. The study aims to assess students' knowledge of COVID-19 transmission methods, symptoms, and preventive measures, as well as their attitudes and practices towards the disease. The findings are presented in a descriptive manner, with statistical analyses conducted to identify significant differences. Overall, the study provides insights into the students' knowledge and behaviors related to COVID-19 and emphasizes the role of media and education programs in improving awareness.

1. The abstract provides a clear overview of the study's background, methods, and findings. Please include years after 17-20 in the results section.
2. The introduction effectively presents the significance of studying students' knowledge, attitudes, and practices during the COVID-19 pandemic. It establishes the context and highlights the potential impact of lockdown measures on students' responses to preventive measures.
3. The methods section describes the study location, sampling design, data collection, and statistical analysis. However, it lacks details on how the online self-structured questionnaire was developed and validated. Providing information on the questionnaire's content validity and reliability would enhance the rigor of the study. Furthermore, the authors have mentioned that the written informed consent was obtained. For the transparency and reproducibility of the methods authors are requested to provide how the written informed consent was obtained. The authors state that the participants were aged from 17-20 years. The authors need to detail who provided the consent for those less than 18 years.
4. The results section presents the findings in a comprehensive manner, using tables and figures to display the participants' knowledge, attitudes, and practices before and after the lockdown.
5. The discussion section provides an in-depth interpretation of the findings and relates them to existing literature. It highlights the importance of media, social relationships, and education programs in shaping students' knowledge and behaviors. The authors should provide limitations of the study, such as the small sample size, focus on a single university, exclusion of female participants etc. The study chooses random participants both for pre-lockdown and post-lockdown which would not reflect individual’s Covid-19 knowledge, attitude and practice assessment.
6. The manuscript adheres to the IMRAD structure and follows a logical flow from introduction to conclusion. The language is generally clear and concise, although there are occasional grammatical errors that could be addressed during proofreading.

Is the work clearly and accurately presented and does it cite the current literature?

Yes

If applicable, is the statistical analysis and its interpretation appropriate?

I cannot comment. A qualified statistician is required.

Are all the source data underlying the results available to ensure full reproducibility?

Yes

Is the study design appropriate and is the work technically sound?

Yes

Are the conclusions drawn adequately supported by the results?

Yes

Are sufficient details of methods and analysis provided to allow replication by others?

No

Reviewer Expertise:

Forensic Medicine & Toxicology, and broad areas of interest are injuries, violence, trauma, abuse, medical ethics, medical education, forensic pathology, forensic anthropology, criminal law, forensic psychiatry, etc.

I confirm that I have read this submission and believe that I have an appropriate level of expertise to confirm that it is of an acceptable scientific standard, however I have significant reservations, as outlined above.

# Response

**Published:** 2023-07-07

**Authors:** Mahmoud Berekaa

**Affiliations:** College of Public Health, Imam Abdulrahman Bin Faisal University, Saudi Arabia

**First, as author of the manuscript, I appreciate valuable comments and suggestions highlighted by the reviewer. Kindly find point-by-point response to reviewer’s comments:**

 1. The abstract provides a clear overview of the study's background, methods, and findings. Please include years after 17-20 in the results section.

**Reply:** Thanks for your comment, done 

 2. The introduction effectively presents the significance of studying students' knowledge, attitudes, and practices during the COVID-19 pandemic. It establishes the context and highlights the potential impact of lockdown measures on students' responses to preventive measures.

**Reply:** Thanks for your comment 

 3. The methods section describes the study location, sampling design, data collection, and statistical analysis. However, it lacks details on how the online self-structured questionnaire was developed and validated. Providing information on the questionnaire's content validity and reliability would enhance the rigor of the study. Furthermore, the authors have mentioned that the written informed consent was obtained. For the transparency and reproducibility of the methods authors are requested to provide how the written informed consent was obtained. The authors state that the participants were aged from 17-20 years. The authors need to detail who provided the consent for those less than 18 years.

**Reply:** As response to the mentioned comments, the following modifications and enrichment data provided:

- The IAU QuestionPro platform was used for data collection. An Arabic self-structured online questionnaire was developed and subjected to face as well as content validation by three public health experts. Before randomly distributed to students, the reliability of the questionnaire was performed using Cronbach’s alpha test, which was found to be moderate (0.60), but mostly acceptable. The same questionnaire was used to assess knowledge levels and attitudes both pre- and post-lockdown.

 

- Eligible students should agree on written and verbal consent before admission of the survey. Others unwilling to participate or unable to understand the content of the questionnaires are excluded and have the right to leave the survey at any time.

 

- On the other hand, we consider that preparatory year students in IAU with the age of 17 are targeted during any public health awareness program in IAU and through ministry of health and other governmental agencies especially during the study period. 

 4. The results section presents the findings in a comprehensive manner, using tables and figures to display the participants' knowledge, attitudes, and practices before and after the lockdown.

**Reply:** Thanks for your comment.

 5. The discussion section provides an in-depth interpretation of the findings and relates them to existing literature. It highlights the importance of media, social relationships, and education programs in shaping students' knowledge and behaviors.

**Reply:** Thanks for your comment.

 6. The authors should provide limitations of the study, such as the small sample size, focus on a single university, exclusion of female participants etc.

**Reply:** The following paragraph addressing the major limitations that should be considered during interpretation of the results was added.

 “On the other hand, there are some limitations that should be considered during interpretation of the results. First, the study focuses on small student number from IAU university, thus cannot be generalized. Second, the questionnaire was only available for male students, with exclusion of female participants, therefore there is a potential for participation bias. Finally, the study chooses random participants both for pre-lockdown and post-lockdown which would not reflect individual’s Covid-19 knowledge, attitude, and practice assessment”

 7. The manuscript adheres to the IMRAD structure and follows a logical flow from introduction to conclusion. The language is generally clear and concise, although there are occasional grammatical errors that could be addressed during proofreading.

**Reply:** the manuscript subjected to proofreading before submission.

- **Is the work clearly and accurately presented and does it cite the current literature?**

Yes

**Reply:** thanks

- **Is the study design appropriate and is the work technically sound?**

Yes

**Reply:** thanks

- **Are sufficient details of methods and analysis provided to allow replication by others?**

No

**Reply:** more details on methods and analysis section provided to allow replication by others.

  

- **Are all the source data underlying the results available to ensure full reproducibility?**

Yes

**Reply:** thanks

- **Are the conclusions drawn adequately supported by the results?**

Yes

**Reply:** thanks

# Peer review: Reviewer response for version 1

**DOI:** 10.5256/f1000research.122528.r149902

**Published:** 2022-09-13

**Authors:** Walyeldin Elfakey

**Affiliations:** 1 University of Bahri, Alkadroo, Sudan

The author studied the student's knowledge, attitude and practices (KAP) towards SARS-CoV-2 outbreak. They compared between the KAP before and after the lockdown.

 Generally, the study is good apart from some points that I highlighted in the following report.

**Title: **Is good and reflects the idea of the study.

**Abstract: **Acceptable.

**Introduction**: Concise and summarizes the relevant literature.

**Methods: **

1. Baseline data pertaining to the participants’ pre-lockdown COVID-19 awareness and practices was gathered – what was the method of gathering this baseline data which reflects the awareness and practices pre-lockdown? This point needs clarification.
2. The author should describe the method of randomization used.
3. The study includes males only, is there any female section in the university, if so why were females not included and there any impact of gender variation in the results? As a report from Saudi Arabia with the largest study that included 3388 participants concluded (However, the results showed that men have less knowledge, less optimistic attitudes, and less good practice toward COVID-19, than women).[Ref-1]  Another study from Bangladesh stated (More frequent prevention practice factors were associated with female sex, older age, higher education, family income > 30,000 BDT, urban area residence, and having more positive attitudes).[Ref-2]

**Results:**

1. The number of household members were categorized in 3 groups 3-5, 6-8, and more than 9, what was the bases behind these categories and why these numbers, I couldn’t find a justification?
2. The author stated (while other methods, such as runny nose, diarrhea, and pain, were identified incorrectly) as this is not correct early reports that describe COVID-19 included these symptoms (see https://www.cdc.gov/coronavirus/2019-ncov/symptoms-testing/symptoms.html).

**Discussion: **Good and acceptable.

**Conclusion: **The opening statement was (The frequent variation in the COVID-19 genome has led to the requirement of different vaccine types, reflecting the importance of preventive measures in mitigating the risks associated with COVID-19 infection). I did not find any relation between this conclusion and this study.

Is the work clearly and accurately presented and does it cite the current literature?

Yes

If applicable, is the statistical analysis and its interpretation appropriate?

Yes

Are all the source data underlying the results available to ensure full reproducibility?

No source data required

Is the study design appropriate and is the work technically sound?

Yes

Are the conclusions drawn adequately supported by the results?

Yes

Are sufficient details of methods and analysis provided to allow replication by others?

Partly

Reviewer Expertise:

Pediatrics and Child Health, Infectious Diseases, Medical Education

I confirm that I have read this submission and believe that I have an appropriate level of expertise to confirm that it is of an acceptable scientific standard.

## References

[1] : Knowledge, Attitude and Practice Toward COVID-19 Among the Public in the Kingdom of Saudi Arabia: A Cross-Sectional Study. *Front Public Health* .2020;8: 10.3389/fpubh.2020.00217 217 10.3389/fpubh.2020.00217 32574300PMC7266869

[2] : Knowledge, attitude, and practice regarding COVID-19 outbreak in Bangladesh: An online-based cross-sectional study. *PLoS One* .2020;15(10) : 10.1371/journal.pone.0239254 e0239254 10.1371/journal.pone.0239254 33035219PMC7546509
